# Analysis of Morphological and Morphometric Changes in a Parenchymal Tissue after the Radiofrequency Ablation Procedure

**DOI:** 10.3390/medicina59040702

**Published:** 2023-04-03

**Authors:** Darijus Skaudickas, Gintautas Vaitiekaitis, Julius Liobikas, Aldona Gružienė, Marcel Abras, Gita Gersone, Aleksandras Vitkus, Sigita Kerzienė, Greta Undžytė, Vincentas Veikutis, Artūras Kašauskas, Armuntas Baginskas, Algis Noreika

**Affiliations:** 1 Medical Academy, Lithuanian University of Health Sciences, LT50161 Kaunas, Lithuania; 2 Department of Internal Medicine, Nicolae Testemitanu State University of Medicine and Pharmacy, MD2004 Chisinau, Moldova; 3 Department of Human Physiology and Biochemistry, Rīga Stradiņš University, LV-1007 Riga, Latvia; 4 Veterinary Academy, Lithuanian University of Health Sciences, LT47181 Kaunas, Lithuania

**Keywords:** radiofrequency ablation, prostate, histology of prostate zones, morphology, electrode

## Abstract

*Background and Objectives:* Prostate cancer is on the rise in the European Union, and radiofrequency ablation (RFA) is one of the minimally invasive treatment options used for its treatment. Therefore, the aim of this study was to investigate and analyze the effects of RFA on prostate tissues. *Materials and Methods:* A standard prostate RFA procedure was performed on 13 non-purebred dogs in three sessions: no cooling (NC), cooling with a 0.1% NaCl solution (C.01), and cooling using a 0.9% NaCl solution (C.09). Microtome-cut 2–3 µm sections of prostate samples were stained with hematoxylin and eosin and further examined. *Results:* A histopathologic evaluation identified four zones of exposure: direct, application, necrosis, and transitional, as the damage on tissues decreased going further from the ablation site. The areas and perimeters of these zones were calculated, and geometric shapes of ablative lesions were evaluated using the quotient formula. Areas and perimeters of prostate tissue lesions in the NC and C.09 sessions were of similar size, whereas those found in C.01 were statistically significantly smaller. Lesions observed in session C.01 were of the most regular geometric shape, while the most irregular ones were found in session C.09. The shapes of lesions closest to the ablation electrode were the most irregular, becoming more regular the further away from the electrode they were. *Conclusions:* Prostate RFA leads to tissue damage with distinct morphological zones. Notably, the prostate lesions were the smallest and the most regular in shape after RFA procedures using the 0.1% NaCl cooling solution. It can be argued that smaller ablation sites may result in smaller scars, thus allowing for faster tissue healing if the blood flow and innervation at the ablation site are not compromised.

## 1. Introduction

As studies show, there were 3.91 million new cancer cases diagnosed in the European Union (EU) in 2018. The most common types of cancer were women’s breast cancer, colorectal cancer, lung cancer, and prostate cancer [1,2]. The estimated number of new cancer cases was approximately 1.6 million in men and 1.4 million in women. In the same year, 790,000 men and 620,000 women died because of oncological diseases [3]. In addition, prostate cancer ranks second after lung cancer in several eastern EU countries [2,4]. For instance, nearly 3500 Lithuanian men are diagnosed with prostate cancer each year, which is far above the average in other EU countries [5]. Early diagnosis and treatment of prostate cancer contribute to beneficial treatment results and survival rates. There are many methods of treating this disease, the choice of which depends on the histology of the tumor, its malignancy, the level of prostate specific antigen (PSA), the stage of the disease, etc. [6]. A possible treatment option is radiofrequency ablation (RFA), which has been used for more than a decade to treat prostate diseases (benign prostatic hyperplasia or cancer) [7]. RFA is one of several medical ablation methods such as cryoablation, microwave ablation, and ablation by laser or by ethanol injections, which is based on the application of medium frequency (350–500 kHz) AC to the desired tissue [7]. A high temperature generated during the procedure affects the histological structure of the targeted tissue. Moreover, RFA can be used alone or with other treatments (surgery, chemotherapy, radiation therapy) to limit or destroy abnormal cells. This stops the course of the oncological disease and improves the patient’s quality of life. RFA is also used in the cancer treatment of other parenchymal organs such as the liver, lungs, and kidney [7,8]. It is worth noting that RFA affects various tissues differently [9]. For instance, RFA experiments on canine striated muscles demonstrated that tissue damage around the ablation electrode formed differently shaped zones [10]. It was suggested that the differences were caused by the inhomogeneity of muscular tissue and were due to the anisotropic electrical and thermal conductivity of striated muscle. Thus, the striated muscles have different values of electrical and thermal conductivity along the fibrils and across the fibrils [9,10]. However, the prostate gland, as a parenchymal organ, consists of two different tissues, namely, the gland and the stroma, which are homogeneous in all directions for both electrical and thermal conductivity [11]. Moreover, the ultrastructural elements are arranged in a highly structured network of collagen fibers.

The main advantage of RFA treatment is the effective destruction of tissues reached by ablation electrodes at various organ sites in the body. RFA treatment of other parenchymal organs, such as the liver [12,13], has shown the importance of this option in the case of low liver function reserve, as it spares the parenchymal tissue of the organ [14]. However, ablated tissues have different morphological structures and are entwined with various nerve plexuses and blood vessels. Therefore, it is important to know the shape and size of the tissue damage after the RFA procedure. In addition, faster tissue healing would be expected if the blood flow and innervation were not impeded at the ablation site. Knowing how ablative parenchymal (prostate) tissue lesions are formed after RFA would facilitate the selection of the appropriate duration and ablative power of the RFA procedure.

Therefore, the aim of the present work was to evaluate the areas and geometrical shapes of prostate parenchymal tissue damage formed during the RFA procedure under different cooling conditions (no cooling and cooling with different concentrations of saline solution), as well as the influence of the nature of cooling on the variation in these parameters. The experiments were carried out on dog prostate tissues because the dog prostate is the closest in structure to that of humans, just with less stoma. Thus, the RFA procedure resulted in morphological tissue damage, and four distinct zones were identified. Notably, tissue damage at the ablation site was the smallest and of the most regular shape when the RFA procedure was performed with a 0.1% NaCl cooling solution, as compared to the RFA procedure without cooling or after the application of a 0.9% NaCl cooling solution. Thus, it can be hypothesized that smaller ablation sites would result in smaller scars and would result in faster tissue healing, if the blood flow and innervation were not compromised at the ablation site.

## 2. Materials and Methods

### 2.1. Animals

The RFA experiments on prostates of 13 non-purebred dogs were performed in the Large Animal Clinic at Veterinary Academy, LUHS. The permission for study No 0027/2001 was granted by the Bioethics Center, LUHS. The weight of dogs varied from 10 to 14 ± 0.05 kg. For the anesthesia, midazolam (bolus, 25 mg; infusion 3–6 mg/h) and ketamine (bolus, 750 mg; infusion 100–200 mg/h) were used. The dogs were euthanized 4 to 6 h after surgery and RFA; the prostate was removed and histological samples were taken.

### 2.2. Radiofrequency Ablation Procedure

The standard RFA procedure developed for parenchymal tissues [15] was adapted for the present study. Briefly, RFA was conducted on each dog in three sessions. In the first RFA session (NC), the procedure was performed without electrode cooling. In the second RFA session (C.01), a cooling solution of 0.1% NaCl at 20 °C was infused at the rate of 16 mm/min during 30 s RFA, while keeping RFA power 40 W, frequency 500 kHz, and impedance from 110 to 210 Ω. For the third RFA session (C.09), all the conditions were kept the same as for the second one, except that the cooling solution was 0.9% NaCl. Two different concentrations of NaCl were used because solutions of such concentrations are commonly used in clinical practice. A typical ablation catheter with a 4 mm length and 2 mm diameter tip Biosense Webster electrode (Johnson & Johnson Medical Devices, Irvine, CA, USA) was used in the study [11].

### 2.3. Histological Analysis

Histological examinations were carried out at the Department of Histology and Embryology, Medical Academy, LUHS. Microtome-cut 2–3 µm sections of prostate samples were stained with hematoxylin and eosin. To calculate prostate lesion areas (S) and perimeters (P), there were 160, 120, and 100 histological preparations in experimental sessions NC, C.01, and C.09, respectively. The micro-images were taken using a computerized microscope OLYMPUS BX 40 (Vilnius, Lithuania) with a resolution of 2080 × 1544 pixels. Given the microscopic magnification of the image, the pixel size was 3.45 × 3.45 μm. Analysis and processing of histological preparations were performed with Image J, Microvision 1.1, and Cell Sens Dimensions 2010 software.

### 2.4. Mathematical Analysis

The quotient calculation formula was used to estimate the geometric shape of the ablation site. A quotient is a ratio between the length of the principal axis and the maximum orthogonal width to the principal axis, calculated by the formula $P_{2A}=\frac{P^{2}}{4\pi A}$, where *P* is the perimeter and *A* is the area. The object is circular if the coefficient is 1. The higher the coefficient, the less the shape of the object resembles a circle [15]. 

### 2.5. Statistical Analysis

Statistical analysis of the data was performed using a statistical package IBM SPSS Statistics 25. Since the Kolmogorov–Smirnov test showed that not all groups had a normal distribution of data, parametric and non-parametric analyses were applied. To compare the properties of the investigated groups, their averages and deviations were calculated. ANOVA with Bonferroni post hoc criterion was used to assess differences between the sessions. To express the differences between the sessions, the medians and their 95% confidence intervals were calculated, and a graphical comparison of distributions was presented. A non-parametric Kruskal–Wallis test was used to assess the differences between the sessions. Differences were considered statistically significant when *p* < 0.05.

## 3. Results

**Figure 1:**
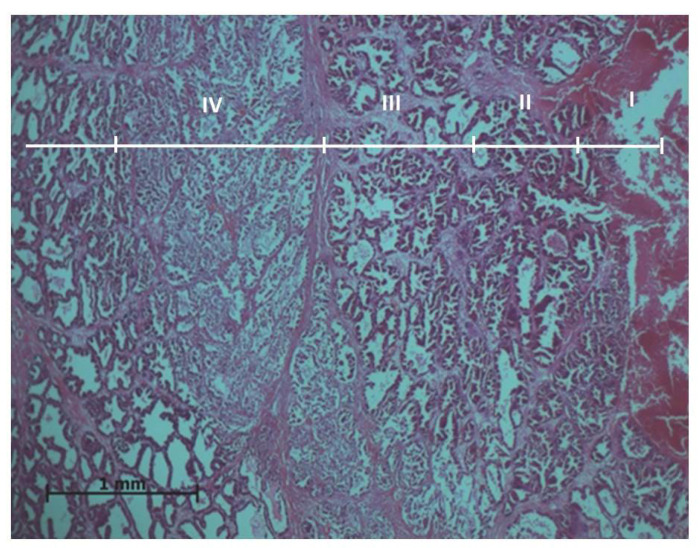
General histologic image of the prostate after ablation: I—ablation site; II—application zone; III—necrosis zone; IV—transition zone. Microtome-cut sections of prostate samples were stained with hematoxylin and eosin.

Experimental results showed that ablation effects on the prostate tissues vary going further from the active electrode. Thus, the histopathological evaluation of the prostate samples resulted in four exposure zones (Figure 1).

**Figure 2:**
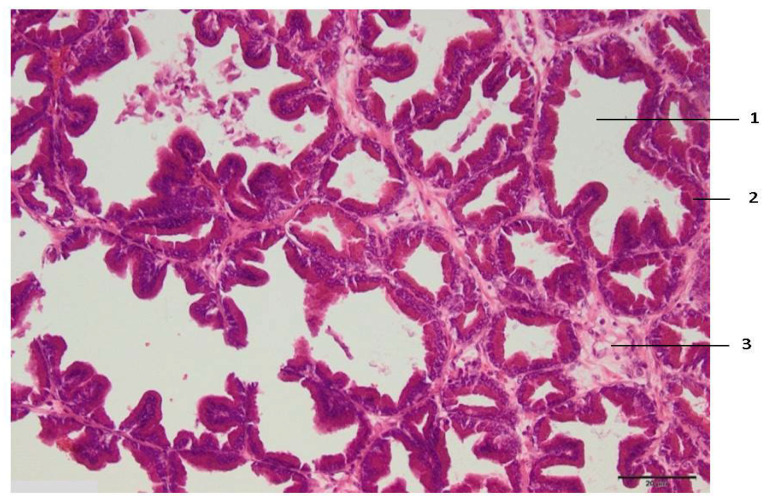
Histologic zone II of the prostate lesion: 1—alveolar gland of the prostate; 2—secretory epithelium with dark pycnotic nuclei; 3—connective tissue edema. Microtome-cut sections of prostate samples were stained with hematoxylin and eosin.

Zone I makes direct contact with the electrode and is always larger than the electrode tip area. Zone II is the application zone adjacent to the thermal electrode (Figure 2). Acinar prostate epithelial cells are in a normal shape, with pronounced eosinophilic cytoplasm and dark pycnotic nuclei (onset of necrosis). Very prominent connective tissue edema and non-striated muscle cells with degenerative lesions are observed.

**Figure 3:**
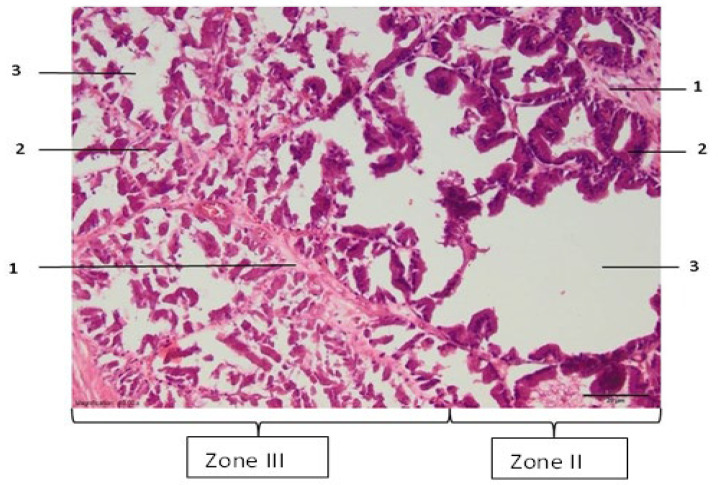
Zone III of the prostate lesion: 1—stroma; 2—glandular epithelium; 3—prostate alveolus. Microtome-cut sections of prostate samples were stained with hematoxylin and eosin.

In zone III, which can be relatively called the necrosis zone, the tissues are affected by coagulative necrosis but retain their shape; however, in our case, it is not fully formed because the gland boundaries are preserved, although with desquamated epithelium cells. The cytoplasm of cells is filled with large granules visualized with the dye eosin. In addition, the nuclei of such cells are dense and compact or even invisible, and necrotic epithelial cells without nuclei predominate. Edema and slight extravasation of red blood cells are observed in the stroma, as well as degeneration and necrosis of non-striated muscle cells (Figure 3). Such an effect could be explained by the fact that the glandular epithelium is more sensitive than the stroma, which consists of muscular and connective tissue cells. The normal glow of collagen fibers is visible under polarizing light.

**Figure 4:**
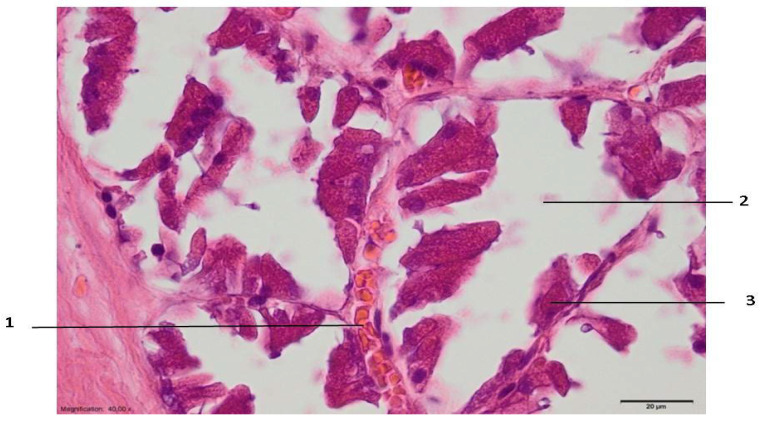
Zone IV of the prostate lesion: 1—dilated blood vessels; 2—prostate epithelium; 3—eosinophilia of secretory cell cytoplasm. Microtome-cut sections of prostate samples were stained with hematoxylin and eosin.

Zone IV is a transitional zone. Lower eosinophilia of the cytoplasm is observed, with less pronounced pyknosis of the nuclei than in the preceding areas. Stromal edema and degeneration of non-striated muscles are also observed. This damage zone extends beyond the lobe, includes the interlobular tissue, and penetrates the adjacent lobes (Figure 4).

**Figure 5:**
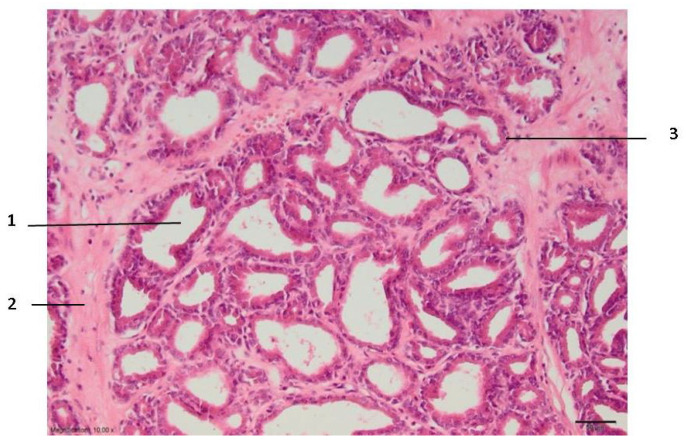
Intact zone of the prostate. Normal tissue structure: 1—glandular epithelium; 2—stroma; 3—prostate alveolus. Microtome-cut sections of prostate samples were stained with hematoxylin and eosin.

Beyond the transition zone, the observed intact zone is composed of normal-looking epithelium and stroma. Under the polarizing microscope, the normal glow of collagen fibers is visible in all zones (Figure 5).

According to the character and severity of tissue damage following the RFA procedure for prostate parenchyma in all three experimental sessions (NC, C.01, and C. 0.9), lesions were divided into four zones with areas S-I, S-II, S-III, and S-IV, and perimeters P-I, P-II, P-III, and P-IV, respectively. Thus, the averages of the RFA tissue damage areas (S) and perimeters (P) in three experimental sessions C.09, C.01, and NC are shown in Table 1.

The biggest average area S-IV was found in session NC, while the average area S-IV was statistically significantly smaller in session C.01. There was no statistically significant difference between average areas S-IV in sessions NC and C.09. The biggest average area of lesion S-III was found in session C.09, and the smallest in session C.01. In session C.01, the average area S-III was statistically significantly smaller compared to sessions C.09 and NC. Average areas S-III in sessions C.09 and NC were not statistically significantly different. The biggest average area S-II was found in session C.09 and the lowest in session C.01.

The average area of lesion S-I was the biggest in session C.09 and the smallest in session C.01. In sessions NC and C.01, the average area of lesion S-I was statistically significantly smaller than in session C.09. There was no statistically significant difference between average areas S-I in sessions C.01 and NC.

When analyzing tissue damage perimeters, we found that lesion perimeters were statistically significantly larger in session C.09 than in sessions C.01 and NC.

The average of P-III in session C.09 was the highest and statistically significantly lower in session C.01. Moreover, the average of P-II was the highest in session C.09 and the lowest in session C.01. There was no statistically significant difference between P-II in sessions C.01 and NC. Comparing the length of P-II, a statistically significant difference between sessions C.09 and C.01 was observed, as well as between sessions C.09 and NC. Whereas C.01 and NC did not show a statistically significant difference. It was impossible to compare the P-I between sessions because a normal distribution was obtained only in the session C.01. Since the areas and perimeters were not normally distributed in all groups, a non-parametric analysis was additionally applied to assess possible differences between the groups.

Thus, it was found that the smallest average lesion areas were in experimental session C.01. The lesions in experimental sessions C.09 and NC were of similar size, and in all zones, were significantly larger than the damage areas produced during experimental session C.01 (see Table 1).

**Figure 6:**
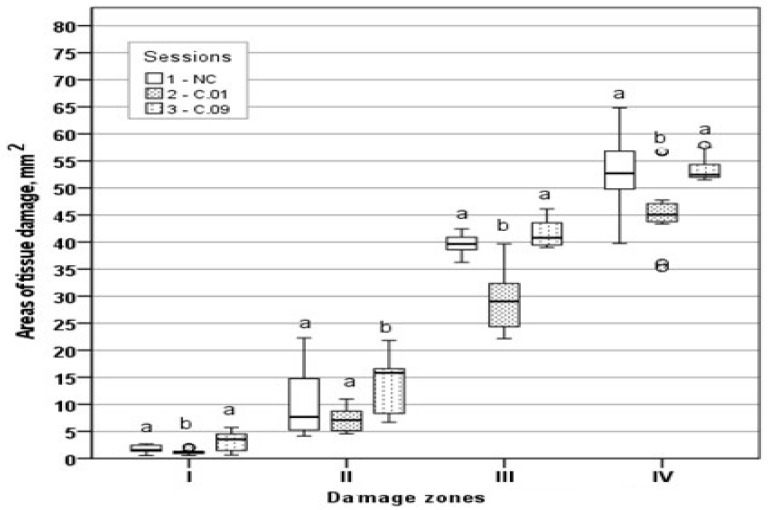
Graphical comparison of tissue damage area distributions by zones. a, b—the distributions differed statistically significantly as found by a non-parametric Kruskal–Wallis test.

**Figure 7:**
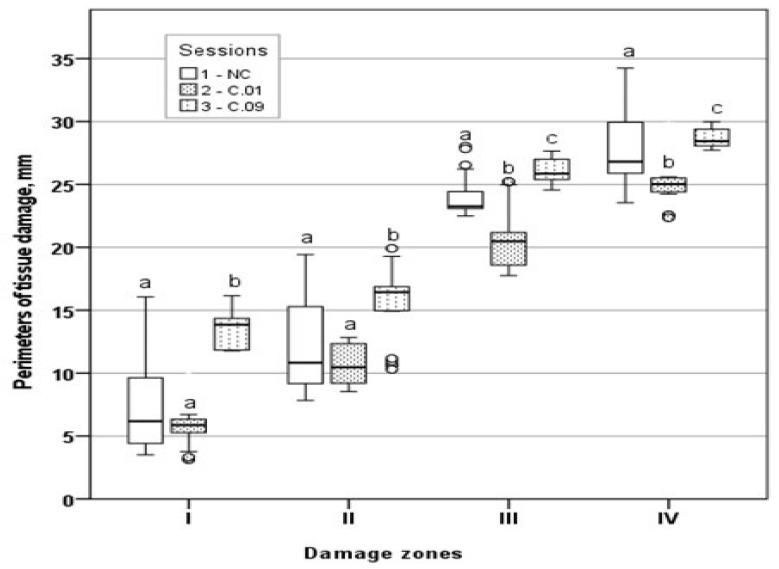
Graphical comparison of tissue damage perimeter distributions by zones. a, b, c—the distributions differed statistically significantly as found by a non-parametric Kruskal–Wallis test.

Moreover, to better express the differences among sessions, the areas and perimeters of tissue damage zones were calculated as their medians and their 95% confidence intervals, which are shown in Table 2 and Table 3, respectively. Tissue damage area distribution and tissue damage perimeter distribution by zones are presented in Figure 6 and Figure 7, respectively.

Thus, the non-parametric analysis confirmed the differences expressed in means. In all zones, both the highest mean area and the highest median area were found in the C.09 session (Table 2). In zones III and IV, the areas of the NC, C.01, and C.09 sessions differed statistically significantly (*p* < 0.05). In zone II, there were statistically significant differences between the C.09 session and NC (*p* < 0.05) and between the C.09 and C.01 sessions (*p* < 0.05). The lowest median was in the C.01 session and it was statistically significantly different from the NC and C.09 sessions in zone I. When comparing both the averages and medians of the session areas, statistically significant differences between the C.09 and C.01 sessions were found in all zones.

In all zones, for both area and perimeter, the highest median was found in session C.09. The perimeters of the C.09 group were statistically significantly (*p* < 0.05) different from both C.01 and NC in all zones. The median perimeters of the NC and C.01 sessions did not differ in zones I and II, although the amplitude of variation in the perimeter values in the NC session (in zone I, 3.52–16.06; in zone II, 7.83–19.43) was much higher than in the C.01 session (in zone I, 3.14–10.33; in zone II, 8.55–12.83). Thus, both parametric and non-parametric analyses revealed the same differences between the compared sessions.

**Figure 8:**
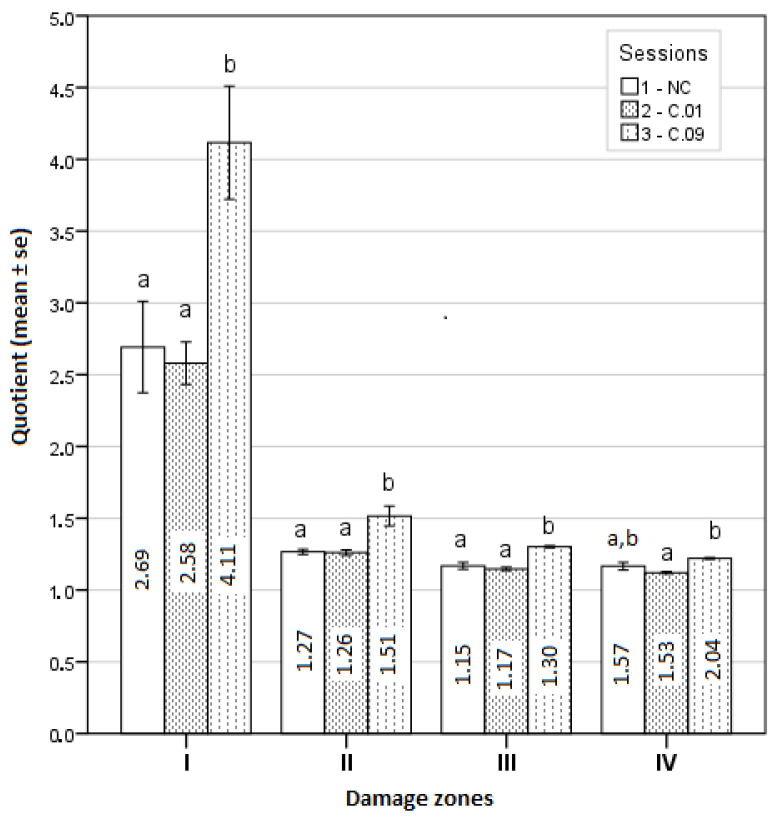
Graphical comparison of damage zone quotients. a, b—the means differed statistically significantly as found by Bonferroni test.

The geometric shapes of lesion zones were evaluated as well, and the calculated averages of quotients for all four zones are presented in Figure 8.

Zone IV was found to have the most regular geometric shape, i.e., the most similar to a circle in session C.01, and the least regular in session C.09. The difference between the means of these quotients was statistically significant (*p* < 0.05). The geometric shape of zone III was the most regular in the NC experiment and the most irregular in session C.09, and the difference between these quotient averages was statistically significant. Moreover, the geometric shapes of zone II and zone I were the most regular in experimental session C.01 and the least regular in session C.09. The differences between these quotient averages were statistically significant (see Figure 8).

## 4. Discussion

In this experimental work, the effect of different RFA treatments (without cooling and with cooling by two NaCl salt solutions) on prostate morphological changes in the formed tissue damage zones (areas and perimeters) was evaluated and determined. The areas and perimeters of damage zones were also assessed, calculated, and compared. Based on this strategy, the four zones of histopathological tissue damage were identified (see Figure 1) as: zone I, which was in direct contact with the electrode; zone II (the application zone) that was adjacent to the thermal electrode (Figure 2); zone III was the necrosis zone (Figure 3); and zone IV was the transition zone (Figure 4). The latter tissue damage area extends beyond the lobe boundaries. The intact zone was observed around the transition zone and consisted of normal epithelium and stroma (see Figure 1 and Figure 5). Moreover, the ablation lesions formed following RFA in the prostate were similar to the lesions formed after RFA in another parenchymal tissue, the liver [16]. Thus, it was observed [16] that after the RFA procedure zones of liver tissue damage were formed, these were identified as the central necrosis zone, the hemorrhagic marginal, and the transitional zones. It has been reported [17] that after the ablation of the liver, hemorrhages with lymphocytic infiltrates occur in the tissue surrounding the necrosis zone, much like in the prostatic parenchyma after ablation procedures.

Moreover, according to [18], the most complex biological processes of the thermal effects of RFA occur in the transitional zone of hepatic tissue injury. This is where the initial phase of cell death begins, marked by a loss of enzymatic activity, characterized by a disruption of cell membranes and mitochondrial functions, with continued degradation of nucleic acids and the cytoskeleton. These processes are also reported in publications of other authors [18,19,20,21]. Notably, similar changes at the morphological level were also observed in the present work (see Figure 4). However, the extent of tissue damage depends on the applied ablation energy, duration of thermal exposure and thermal sensitivity, and heat conduction of the cells, and the thermal properties that vary between organs and tissues [22].

Moreover, the obtained results indicate that ablation tissue damage in all four zones was the lowest when the RFA procedure was performed with the 0.1% NaCl cooling solution as in session C.01 (see Table 1). This is important to know when selecting an appropriate solution and its concentration to cool tissues during RFA procedures. Thus, the postoperative ablation sites could be expected to form smaller scars, which might involve existing major structures: nerve plexuses, vascular networks, and lymphatic vessels. Faster tissue healing would be ensured if the blood flow and innervation were not obstructed at the ablation site. Knowing how ablative lesions of parenchymal (prostate) tissues (morphologic changes, zones, areas, perimeters, geometric shapes) are formed after cooled RFA, it would be possible to predict cancer tissue regeneration and the probability of relapses, and to select the appropriate RFA procedure parameters, such as duration and ablative power.

As stated before, a quotient parameter was used to estimate the geometry of the tissue damage. Since some parameter values were calculated from a normal distribution and some from a non-normal distribution, it was difficult to estimate the differences between geometric shapes from the averages. To simplify the representation of the results, it was assumed that all parameters were in accordance with the law of normal distribution, and a graphical representation of quotients is given in Figure 8. Thus, it can be suggested that moving away from the center, the geometric shape of the lesion approaches a circle shape. The first damage zone is the most irregular, while the other three damage zones resemble a regular circle. Furthermore, the most irregular geometric shapes of all four damage zones were found in the experimental session C.09. The most irregular geometric shape of damaged tissue in the first zone near the electrode is possibly related to the ablation power and the exposure time of the ablation procedure. Therefore, it could be recommended to start with a lower ablation power and a shorter duration. In addition, a higher ablation power and a longer RFA duration can also have an effect on the further success of the ablation procedure, resulting in deeper tissue damage. A less irregular geometrical shape of damaged tissue was formed using the 0.1% NaCl solution for cooling. This could be useful to know for further experimental studies, and later, in clinical practice.

## 5. Conclusions

Experiments have shown that morphological tissue damage that follows RFA of the prostate forms four distinct zones. When performing RFA procedures with a 0.1% NaCl cooling solution, tissue damage at the ablation site is the smallest as compared to the RFA procedure without cooling or after the application of a 0.9% NaCl cooling solution. The resulting geometric shape of the damaged tissue is the most regular in experimental session C.01. Thus, it can be suggested that smaller ablation sites can be expected to form smaller scars, and consequently, a faster tissue healing is ensured if the blood flow and innervation are not obstructed at the ablation site.

## Figures and Tables

**Table 1:** The average areas (S) and perimeters (P) of all four damage zones in three experimental sessions.

Session	*n*	Area (S)				Perimeter (P)			
		I	II	III	IV	I	II	III	IV
NC	160	1.74 ± 0.11 **a**	10.25 ± 0.95 **a**	39.58 ± 0.25 **a**	53.80 ± 0.86 **a**	7.45 ± 0.61 **a**	12.30 ± 0.59 **a**	24.07 ± 0.28 **a**	28.04 ± 0.49 **a**
C.01	120	1.18 ± 0.08 **a**	7.31 ± 0.37 **b**	29.60 ± 1.04 **b**	45.61 ± 1.17 **b**	6.16 ± 0.38 **a**	10.65 ± 0.28 **a**	20.58 ± 0.42 **b**	25.29 ± 0.38 **b**
C.09	100	3.15 ± 0.37 **b**	13.8 ± 1.10 **c**	41.69 ± 0.52 **a**	53.56 ± 0.44 **a**	11.88 ± 0.89 **b**	15.63 ± 0.56 **b**	26.10 ± 0.19 **c**	28.65 ± 0.15 **a**

a, b, c—different letters in the column mark statistically significantly different mean values (*p* < 0.05, Bonferroni criterion); *n*—number of histological preparations in separate experimental sessions.

**Table 2:** Areas (S) of the tissue damage zones calculated as their medians and confidence intervals.

Session	*n*	Area (S) of Tissue Damage Zone (Median and 95% Confidence Interval)			
		I	II	III	IV
NC	160	1.56 0.86–1.97	7.69 3.15–10.44	39.63 38.83–40.25	52.7 46.17–56.57
C.01	120	1.11 0.90–1.25	7.09 6.28–7.86	29.03 24.81–31.16	45.06 44.38–45.60
C.09	100	3.55 2.90–4.20	15.84 14.45–17.23	40.82 39.44–42.19	52.44 51.88–52.99

*n*—number of histological preparations in separate experimental sessions.

**Table 3:** Perimeters (P) of tissue damage zones, calculated as their medians and confidence intervals.

Session	*n*	Perimeter (P) of the Tissue Damage Zone (Median and 95% Confidence Interval)			
		I	II	III	IV
NC	160	6.17 0.26–9.38	10.83 7.74–12.62	23.25 23.07–23.40	26.81 26.23–27.23
C.01	120	5.87 5.32–6.31	10.46 9.70–11.17	20.48 19.65–21.14	25.02 24.83–25.22
C.09	100	13.85 13.55–14.17	16.44 16.01–16.88	25.85 24.84–26.88	28.44 28.16–28.73

*n*—number of histological preparations in separate experimental sessions.

## Data Availability

Data can be presented upon request from D.S. or G.V.
